# Low Serum 25-Hydroxyvitamin D Levels Are Associated With Hyperandrogenemia in Polycystic Ovary Syndrome: A Cross-Sectional Study

**DOI:** 10.3389/fendo.2022.894935

**Published:** 2022-05-02

**Authors:** Chang Shan, Yu-chen Zhu, Jie Yu, Yi Zhang, Yu-ying Wang, Nan Lu, Jie Cai, Wei Liu, Tao Tao

**Affiliations:** Department of Endocrinology, Renji Hospital, School of Medicine, Shanghai Jiaotong University, Shanghai, China

**Keywords:** vitamin D deficiency, 25(OH)D, polycystic ovary syndrome, hyperandrogenemia, overweight

## Abstract

**Background:**

Increasing evidence suggests a link between vitamin D and polycystic ovary syndrome (PCOS). However, whether vitamin D is related to hyperandrogenemia in PCOS is still inconclusive. The aim of our study is to elucidate the relationship between vitamin D and hyperandrogenemia in women with PCOS in China.

**Methods:**

This is a cross-sectional study including 625 Chinese women with PCOS and 217 controls from January 2016 to June 2020. The anthropometric and biochemical parameters related to 25(OH)D, sex steroids, glucose and lipid profiles were measured.

**Results:**

Serum 25(OH)D levels were lower in women with PCOS than controls (33.99 ± 15.05 vs 36.58 ± 16.49 nmol/L, P = 0.034), especially lower in hyperandrogenic women with PCOS (32.79 ± 14.24 vs 36.21 ± 16.27 nmol/L, P = 0.007). Higher 25(OH)D levels were independently associated with lower risks of hyperandrogenemia after adjusting demographic, metabolic and hormonal confounders (OR = 0.982, 95% CI: 0.969 - 0.995, P = 0.006). Consistent results were observed in subgroup analyses. Among PCOS women with vitamin D deficiency, females with age ≥ 26 years had lower risks of hyperandrogenemia (OR = 0.611, 95% CI = 0.389 - 0.958, P = 0.032), while overweight patients had higher risks of hyperandrogenemia (OR = 2.202, 95% CI = 1.130 - 4.293, P = 0.020) after adjusting multiple confounders.

**Conclusions:**

Our study reported lower vitamin D levels in Chinese women with PCOS, especially in those with hyperandrogenemia. An independent negative correlation between 25(OH)D and hyperandrogenemia was noted in PCOS. For PCOS women with vitamin D deficiency, females that have higher BMI with age < 26 years may be prioritized for hyperandrogenemia assessment.

## Introduction

Polycystic ovary syndrome (PCOS) is a common reproductive endocrine and metabolic disorder in women of childbearing age, with a prevalence of 5% - 10% (1). It is characterized by polycystic ovaries, oligo-amenorrhea, and hyperandrogenism (2). Hyperandrogenism is present in up to 90% of affected women, and mounting evidence suggests that hyperandrogenism is a major pathological mechanism of this syndrome which not only causes ovulation disorders and cutaneous manifestations, but also promotes insulin resistance (IR) and metabolic disturbances (2). However, the etiology and pathophysiology of hyperandrogenism in PCOS remains largely unknown.

Vitamin D is well known for its role in regulating calcium, phosphorus and bone metabolism. Accumulating evidences demonstrate its versatile extra-skeletal functions, including important regulations in metabolic diseases, inflammatory disorders, connective tissue diseases and tumors (3, 4). In addition, present researches have shown that vitamin D may play a role in female reproductive function. Evidence for its modulation of female reproduction physiology are as follows: (1) The nuclear receptor for vitamin D (VDR) and 1α-hydroxylase are expressed in hypothalamus-pituitary-ovary (HPO) axis and other reproductive tissues such as uterus and placenta (5–7); (2) Vitamin D regulates genes responsible for aspects of ovarian, endometrial, and placental function (8–10); (3) Vitamin D plays a regulatory role in sex hormone steroidogenesis (8); (4) Due to its anti-inflammatory and immunomodulatory properties, vitamin D deficiency contributes to the pathogenesis of endometriosis (11).

Observational studies found that 67 - 85% women with PCOS present vitamin D deficiency, and vitamin D deficiency is associated with comorbidities related to PCOS (12–14). Besides, PCOS and vitamin D deficiency share some similar metabolic characteristics based on their close relationships with obesity and IR (15–17). Thus, the association between vitamin D and PCOS has gained more attention. A growing number of evidence suggests that vitamin D may have a link with PCOS-associated manifestations, including ovulatory dysfunctions, hyperandrogenism, IR, dyslipidaemia and metabolic risk factors (4). However, whether vitamin D is related to hyperandrogenism in PCOS is still inconclusive. Inconsistent results have been reported by meta-analyses regarding the effect of vitamin D supplementation on androgens in women with PCOS (18, 19). Therefore, the aim of our study was to elucidate the link between vitamin D and hyperandrogenemia (HA) in Chinese women with PCOS.

## Materials and Methods

### Subjects

This is a cross-sectional study conducted among subjects who referred to the outpatient endocrine clinics of Shanghai Renji hospital from January 2016 to June 2020 with main complains of oligo-menorrhea or amenorrhea, hirsutism, acne and fertility problems. PCOS was diagnosed by an endocrinologist based on the Rotterdam 2003 criteria, with the presence of at least two of the following three components: (1) clinical and/or biochemical signs of hyperandrogenism; (2) oligo-menorrhea or amenorrhea; (3) polycystic ovaries on ultrasound (20). Disorders with similar clinical features were excluded before the diagnosis of PCOS, including 21-hydroxylase-deficient non-classical adrenal hyperplasia, Cushing’s syndrome, hyperprolactinemia, and androgen-secreting neoplasias. Only newly diagnosed patients with PCOS were included into our study. Controls were women with normal ovulation cycles and normal ovarian appearance on ultrasound, without symptoms or signs of hyperandrogenism and/or serum total testosterone levels ≤ 0.6 ng/ml (21). Exclusion criteria for all subjects were thyroid dysfunctions, parathyroid disorders, renal or liver insufficiency, autoimmune diseases and other malabsorption-related disorders. Besides, all subjects had not received any medications or hormonal treatments or vitamin D or multivitamin supplements within 3 months prior to the study inclusion, and had no bypass surgery before the study. Totally, 625 women with PCOS and 217 controls were included.

This study was approved by the Ethical Committees of Renji Hospital and written informed consent was obtained from all subjects. All the study procedures were carried out in accordance with the guidelines of the Declaration of Helsinki for experiments involving humans.

### Anthropometric Measurements

All participants received weight and height measurement with a digital scale and a stadiometer by a specially-assigned person at the initial screening appointment. Body mass index (BMI) was calculated by dividing weight (kg) by the square of height (m^2^). Overweight is defined as BMI 24.0 - 28.0 kg/m^2^ and obesity is defined as BMI ≥ 28 kg/m^2^ according to Chinese overweight/obesity consensus (22). Waist circumference (WC) and hip circumference were measured at midpoint between the lowest rib and the top of iliac crest or at the level of the maximum extension of the hip (21). Waist-hip circumference ratio (WHR) was calculated as the division of WC by hip circumference.

### Blood Biochemistry

All subjects had their fasting blood drawn for laboratory analyses after a 12-h fasting during the 2nd to 5th day of the spontaneous menstrual cycle. Fasting blood samples were collected during a progestin withdrawal bleeding episode if the subject presented amenorrhea for more than three months. The blood samples were stored at 4°C, centrifuged within 1 h and analyzed on the day of blood drawn. As a major reflector of vitamin D reserves, serum 25-hydroxyvitamin D [25(OH)D] levels were measured with an automated electrochemiluminescene immunoassay (ECLIA). The instruments and reagents were from E 170 Modular Analytics (Roche Diagnostics) and the detection range is 10 - 250 nmol/L, with an intra-assay coefficient of variation (CV) of 3.5% - 4.9% and an inter-assay CV of 4.4% - 7.1%. As previously described, vitamin D deficiency is defined as 25(OH)D < 50 nmol/L, insufficiency as 50 - 75 nmol/L, and sufficiency as ≥ 75 nmol/L (16, 23). Biochemical indexes were measured with Roche reagents (D 2400 and E 170 Modular Analytics modules with Roche/Hitachi analyzers; Roche Diagnostics), including alanine aminotransferases (ALT), aspartate aminotransferases (AST), γ-glutamyl transaminase (γ-GT), serum creatinine (Scr), total cholesterol (Tch), triglyceride (TG), low-density lipoprotein (LDL), high-density lipoprotein (HDL), uric acid (UA) and fasting plasma glucose (FPG). Hormones were analyzed by chemiluminescence (Elecsys Auto analyzer, Roche Diagnostics), including insulin, thyroid stimulating hormone (TSH), luteinizing hormone (LH), follicle stimulating hormone (FSH), testosterone (T), sulfated dehydroepiandrosterone (DHEAS), androstenedione (A2), free testosterone (FT), estradiol (E2) and sex hormone binding globulin (SHBG). Besides, high-sensitivity C-reactive protein (hs-CRP) was measured using immunonephelometric assays with a BN-II analyzer (Dade Behring, Deerfield, Germany). Free androgen index (FAI) was calculated as 100* [T (nmol/L)/SHBG (nmol/L)] (24), and HA was defined as FAI ≥ 7 (25). IR was estimated by the homeostasis model assessment of IR (HOMA-IR), which was calculated as [fasting insulin (mIU/L) * fasting glucose (mmol/L)/22.5] (26).

### Statistical Analysis

Statistical analyses were performed with Empowerstats software (http://www.empowerstats.com, X&Y Solutions, Inc., Boston, MA) and SPSS software (version 24.0, SPSS Inc., Chicago, IL, USA). Kolmogorov - Smirnov test was used to examine the distributions of variables. Data were presented as the means ± standard deviation (SD) for continuous variables of normal distribution, median (Q1- Q3) for continuous variables of skewed distribution, and number (%) for categorical variables, respectively. Differences between two groups were compared by independent-sample t-tests. One-way analysis of variance or the Kruskal-Wallis H-test was used for the comparisons of continuous variables of normal or skewed distribution, whereas the Chi-squared test was used for the comparisons of categorical variables. Correlations of HA with metabolic and hormonal parameters were calculated by the Spearman correlation analysis. Multivariable logistic regression models and smooth curve fittings were used to examine the relationship between 25(OH)D and HA. We constructed three models: (1) unadjusted; (2) adjusted for age, BMI, WC, HOMA-IR and LDL; (3) adjusted for age, BMI, WC, HOMA-IR, HbA1c, hs-CRP, ALT, AST, γ-GT, UA, TG, Tch, HDL, LDL, TSH, LH, FSH and E2. P-value < 0.05 was considered statistically significant.

## Results

### Clinical and Biochemical Features of Controls and Women With PCOS

**Table 1** shows the features of 217 controls and 625 women with PCOS. Serum 25(OH)D levels were significantly lower in women with PCOS (33.99 ± 15.05 vs 36.58 ± 16.49 nmol/L, P = 0.034). Notably, vitamin D deficiency was present in 82.95% of controls and 86.24% of women with PCOS, and vitamin D insufficiency was present in 15.21% of controls and 12.16% of women with PCOS.

**Table 1:** Characteristics of controls and women with PCOS.

Variables	Controls (n = 217)	PCOS (n = 625)	P-value
**Age (years)**	28.67 ± 6.27	26.05 ± 5.71	<0.001
**BMI (kg/m^2^)**	25.27 ± 5.84	26.39 ± 5.84	0.015
**WC (cm)**	84.45 ± 14.23	86.84 ± 14.53	0.036
**WHR**	0.87 ± 0.08	0.87 ± 0.08	0.353
**25(OH)D (nmol/L)**	36.58 ± 16.49	33.99 ± 15.05	0.034
** < 50 nmol/L**	180 (82.95%)	539 (86.24%)	
** ≥ 50, < 75 nmol/L**	33 (15.21%)	76 (12.16%)	
** ≥ 75 nmol/L**	4 (1.84%)	10 (1.60%)	
**HbA1c (%)**	5.28 ± 1.14	5.40 ± 1.11	0.161
**HOMA-IR**	1.42 (0.88-2.36)	2.10 (1.23-3.74)	<0.001
**hs-CRP (mg/L)**	0.69 (0.24-2.39)	1.12 (0.40-3.30)	<0.001
**ALT (U/L)**	16.00 (11.00-26.00)	19.00 (12.00-37.00)	0.002
**AST (U/L)**	17.00 (14.00-24.00)	18.10 (15.00-27.00)	0.003
**γ-GT (U/L)**	16.00 (11.84-25.70)	19.80 (13.00-33.00)	<0.001
**Scr (umol/L)**	55.44 ± 9.79	56.05 ± 9.61	0.422
**UA (umol/L)**	299.15 ± 74.33	330.05 ± 82.72	<0.001
**TG (mmol/L)**	0.96 (0.66-1.43)	1.16 (0.79-1.76)	<0.001
**Tch (mmol/L)**	4.58 ± 1.04	4.75 ± 0.94	0.025
**HDL (mmol/L)**	1.47 ± 0.43	1.33 ± 0.38	<0.001
**LDL (mmol/L)**	2.56 ± 0.74	2.80 ± 0.81	<0.001
**TSH (mU/L)**	1.77 (1.14-2.59)	2.11 (1.38-3.17)	0.006
**LH (IU/L)**	5.42 (3.27-9.49)	8.08 (4.46-12.62)	<0.001
**FSH (IU/L)**	6.96 ± 2.77	6.31 ± 2.54	0.002
**E2 (pmol/L)**	203.00 (121.00-300.00)	173.00 (117.00-241.09)	0.014
**DHEAS (ng/ml)**	204.01 ± 92.11	239.81 ± 106.21	<0.001
**A2 (ug/ml)**	2.67 (2.05-3.54)	3.80 (2.85-5.19)	<0.001
**T (nmol/L)**	1.75 ± 0.77	2.31 ± 0.94	<0.001
**FT (pmol/L)**	0.03 (0.02-0.03)	0.05 (0.03-0.06)	<0.001
**FAI**	4.11 (2.86-5.67)	9.16 (5.18-14.70)	<0.001
**SHBG (nmol/L)**	39.00 (29.30-59.00)	24.30 (15.50-38.70)	<0.001

WC, waist circumference; WHR, waist-hip circumference ratio; hs-CRP, high-sensitivity C-reactive protein; ALT, alanine aminotransferases; AST aspartate aminotransferases; γ-GT, γ-glutamyl transaminase; Scr, serum creatinine; UA, uric acid; TG, triglyceride; Tch, total cholesterol; LDL, low-density lipoprotein; HDL, high-density lipoprotein; TSH, thyroid stimulating hormone; LH, luteinizing hormone; FSH, follicle stimulating hormone; E2, estradiol; DHEAS, sulfated dehydroepiandrosterone; A2, androstenedione; T, testosterone; FT, free testosterone; FAI, Free androgen index; SHBG, sex hormone binding globulin.

### Characteristics of Women With PCOS Stratified by the Presence of HA

Women with PCOS were then divided into two groups (non-HA group and HA group) according to their FAI, and their clinical and biochemical characteristics are presented in **Table 2**. As is shown, 219 patients (35.04%) were non-hyperandrogenic while 406 patients (64.96%) were hyperandrogenic. Serum 25(OH)D levels were significantly lower in PCOS women with HA (32.79 ± 14.24 vs 36.21 ± 16.27 nmol/L, P = 0.007).

**Table 2:** Clinical and biochemical features of PCOS with or without HA.

Variables	non-HA (n = 219)	HA (n = 406)	P-value
**Age (years)**	26.69 ± 5.17	25.71 ± 5.95	0.042
**BMI (kg/m^2^)**	22.95 ± 4.78	28.24 ± 5.52	<0.001
**WC (cm)**	78.84 ± 12.32	91.16 ± 13.79	<0.001
**WHR**	0.84 ± 0.07	0.89 ± 0.07	<0.001
**25(OH)D (nmol/L)**	36.21 ± 16.27	32.79 ± 14.24	0.007
**HbA1c (%)**	5.13 ± 0.96	5.55 ± 1.16	<0.001
**HOMA-IR**	1.30 (0.81-2.05)	2.63 (1.65-4.86)	<0.001
**hs-CRP (mg/L)**	0.54 (0.23-1.54)	1.69 (0.64-4.12)	<0.001
**ALT (U/L)**	14.00 (10.00-20.60)	24.00 (14.05-47.80)	<0.001
**AST (U/L)**	17.00 (14.00-20.50)	20.00 (15.00-30.93)	<0.001
**γ-GT (U/L)**	14.80 (11.00-21.80)	24.55 (15.50-41.00)	<0.001
**Scr (umol/L)**	56.74 ± 9.53	55.67 ± 9.64	0.186
**UA (umol/L)**	299.47 ± 67.51	346.55 ± 85.50	<0.001
**TG (mmol/L)**	0.91 (0.65-1.27)	1.35 (0.93-1.98)	<0.001
**Tch (mmol/L)**	4.62 ± 0.90	4.81 ± 0.95	0.015
**HDL (mmol/L)**	1.52 ± 0.41	1.22 ± 0.32	<0.001
**LDL (mmol/L)**	2.60 ± 0.73	2.91 ± 0.83	<0.001
**TSH (mU/L)**	1.98 (1.31-2.89)	2.20 (1.42-3.20)	0.031
**LH (IU/L)**	7.77 (4.13-13.86)	8.45 (4.75-12.55)	0.250
**FSH (IU/L)**	6.52 ± 2.53	6.20 ± 2.54	0.130
**E2 (pmol/L)**	165.00 (106.50-264.50)	179.00 (129.75-240.00)	0.207
**T (nmol/L)**	1.85 ± 0.77	2.55 ± 0.94	<0.001
**FT (pmol/L)**	0.03 ± 0.01	0.06 ± 0.02	<0.001
**FAI**	3.92 (2.60-5.35)	12.41 (9.54-18.26)	<0.001
**SHBG (nmol/L)**	46.60 (35.10-65.70)	18.35 (12.50-24.87)	<0.001
**DHEAS (ng/ml)**	204.93 ± 84.92	258.62 ± 111.74	<0.001
**A2 (ug/ml)**	3.34 (2.50-4.72)	4.08 (3.06-5.40)	<0.001

WC, waist circumference; WHR, waist-hip circumference ratio; hs-CRP, high-sensitivity C-reactive protein; ALT, alanine aminotransferases; AST aspartate aminotransferases; γ-GT, γ-glutamyl transaminase; Scr, serum creatinine; UA, uric acid; TG, triglyceride; Tch, total cholesterol; LDL, low-density lipoprotein; HDL, high-density lipoprotein; TSH, thyroid stimulating hormone; LH, luteinizing hormone; FSH, follicle stimulating hormone; E2, estradiol; T, testosterone; FT, free testosterone; FAI, Free androgen index; SHBG, sex hormone binding globulin; DHEAS, sulfated dehydroepiandrosterone; A2, androstenedione.

### Characteristics of Women With PCOS Stratified by 25(OH)D Tertiles

We then divided women with PCOS into three groups on the basis of their serum 25(OH)D levels. **Table 3** illustrates their clinical and biochemical characteristics. Serum 25(OH)D levels were deficient in 539 women (86.24%), insufficient in 76 women (12.16%) and sufficient in only 10 women (1.60%) with PCOS. Significant differences were observed in FT (P = 0.035) and FAI among groups (P = 0.038). In addition, a borderline difference was observed in SHBG (P = 0.061) and HA (P = 0.064).

**Table 3:** Clinical and biochemical characteristics of women with PCOS stratified by serum 25(OH)D levels.

Variables	25(OH)D level (nmol/L)			P-value	
	< 50 (n = 539)	≥ 50, < 75 (n = 76)	≥ 75 (n = 10)	
**Age (years)**	25.99 ± 5.76	26.24 ± 5.44	27.81 ± 4.98	0.582
**BMI (kg/m^2^)**	26.35 ± 5.79	26.95 ± 6.33	24.06 ± 4.25	0.316
**WC (cm)**	86.97 ± 14.48	86.80 ± 15.52	80.40 ± 7.24	0.367
**WHR**	0.87 ± 0.07	0.87 ± 0.08	0.85 ± 0.06	0.547
**HbA1c (%)**	5.41 ± 1.13	5.38 ± 0.98	5.45 ± 1.44	0.965
**HOMA-IR**	2.15 (1.26-3.73)	2.04 (1.13-3.97)	1.32 (0.84-2.34)	0.268
**hs-CRP (mg/L)**	1.11 (0.41-3.29)	1.37 (0.47-3.50)	0.75 (0.29-2.78)	0.716
**ALT (U/L)**	19.00 (12.00-36.00)	22.00 (12.75-40.00)	14.50 (11.00-24.60)	0.492
**AST (U/L)**	18.00 (15.00-27.00)	21.00 (16.00-28.18)	14.70 (12.50-41.25)	0.191
**γ-GT (U/L)**	19.30 (13.00-32.70)	24.00 (13.78-36.25)	15.50 (13.88-27.90)	0.440
**Scr (umol/L)**	55.72 ± 9.68	57.60 ± 8.51	61.65 ± 11.67	0.050
**UA (umol/L)**	329.72 ± 83.35	328.94 ± 81.35	356.50 ± 56.70	0.594
**TG (mmol/L)**	1.16 (0.78-1.74)	1.17 (0.85-1.80)	1.24 (0.84-1.81)	0.586
**Tch (mmol/L)**	4.74 ± 0.95	4.75 ± 0.84	4.87 ± 0.96	0.918
**HDL (mmol/L)**	1.32 ± 0.38	1.32 ± 0.41	1.42 ± 0.29	0.711
**LDL (mmol/L)**	2.80 ± 0.83	2.79 ± 0.71	2.80 ± 0.83	0.986
**TSH (mU/L)**	2.07 (1.35-3.10)	2.33 (1.43-3.71)	2.30 (0.99)	0.236
**LH (IU/L)**	8.08 (4.40-12.91)	8.25 (4.65-11.20)	7.38 (5.22-8.47)	0.983
**FSH (IU/L)**	6.36 ± 2.57	6.15 ± 2.28	4.87 (2.56-6.36)	0.188
**E2 (pmol/L)**	176.00 (119.50-240.54)	153.50 (98.50-213.25)	205.50 (117.75-471.50)	0.153
**DHEAS (ng/ml)**	238.41 ± 107.09	253.84 ± 102.64	208.30 ± 76.54	0.317
**A2 (ug/ml)**	3.80 (2.83-5.14)	3.85 (3.02-5.41)	4.25 ± 1.68	0.744
**T (nmol/L)**	2.32 ± 0.96	2.27 ± 0.85	2.04 ± 0.53	0.595
**FT (pmol/L)**	0.05 (0.03-0.06)	0.05 (0.03-0.06)	0.03 (0.02-0.04)	0.035
**FAI**	9.43 (5.22-14.72)	9.06 (5.44-14.30)	3.96 (3.16-7.78)	0.038
**SHBG (nmol/L)**	24.50 (15.50-38.45)	22.50 (15.43-37.73)	43.30 (27.43-72.90)	0.061
**HA**				0.064
**No**	185 (34.32%)	27 (35.53%)	7 (70.00%)	
**Yes**	354 (65.68%)	49 (64.47%)	3 (30.00%)	

HA, hyperandrogenemia; WC, waist circumference; WHR, waist-hip circumference ratio; hs-CRP, high-sensitivity C-reactive protein; ALT, alanine aminotransferases; AST aspartate aminotransferases; γ-GT, γ-glutamyl transaminase; Scr, serum creatinine; UA, uric acid; TG, triglyceride; Tch, total cholesterol; LDL, low-density lipoprotein; HDL, high-density lipoprotein; TSH, thyroid stimulating hormone; LH, luteinizing hormone; FSH, follicle stimulating hormone; E2, estradiol; DHEAS, sulfated dehydroepiandrosterone; T, testosterone; A2, androstenedione; FT, free testosterone; FAI, Free androgen index; SHBG, sex hormone binding globulin.

### Association Between Serum 25(OH)D Levels and HA

The data in **Table 4** showed that serum 25(OH)D levels were significantly negatively correlated with HA in women with PCOS (r = - 0.108, P = 0.007). In addition, HA was negatively correlated with age, HDL and SHBG (all P < 0.05), while positively related to BMI, WC, WHR, HbA1c, hs-CRP, TG, Tch, LDL, DHEAS, A2, T, FT and FAI (all P < 0.05) in women with PCOS.

**Table 4:** Correlations between HA and metabolic and hormonal parameters in women with PCOS.

Metabolic Parameters	R	P-value	Hormonal parameters	R	P-value
**Age (years)**	-0.081^*^	0.042	25(OH)D	-0.108^**^	0.007
**BMI (kg/m^2^)**	0.432^**^	<0.001	TSH (mU/L)	0.03	0.451
**WC (cm)**	0.405^**^	<0.001	LH (IU/L)	-0.009	0.818
**WHR**	0.306^**^	<0.001	FSH (IU/L)	-0.061	0.130
**HbA1c (%)**	0.179^**^	<0.001	E2 (pmol/L)	-0.001	0.988
**HOMA-IR**	-0.067	0.097	SHBG (nmol/L)	-0.612^**^	<0.001
**hs-CRP (mg/L)**	0.225^**^	<0.001	DHEAS (ng/ml)	0.241^**^	<0.001
**TG (mmol/L)**	0.233^**^	<0.001	A2 (ug/ml)	0.157^**^	<0.001
**Tch (mmol/L)**	0.097^*^	0.015	T (nmol/L)	0.354^**^	<0.001
**HDL (mmol/L)**	-0.376^**^	<0.001	FT (pmol/L)	0.683^**^	<0.001
**LDL (mmol/L)**	0.186^**^	<0.001	FAI	0.573^**^	<0.001

Significant values are presented: *: P<0.05, **: P<0.001. WC, waist circumference; WHR, waist-hip circumference ratio; hs-CRP, high-sensitivity C-reactive protein; ALT, alanine aminotransferases; AST aspartate aminotransferases; γ-GT, γ-glutamyl transaminase; Scr, serum creatinine; UA, uric acid; TG, triglyceride; Tch, total cholesterol; LDL, low-density lipoprotein; HDL, high-density lipoprotein; TSH, thyroid stimulating hormone; LH, luteinizing hormone; FSH, follicle stimulating hormone; E2, estradiol; DHEAS, sulfated dehydroepiandrosterone; T, testosterone; A2, androstenedione; FT, free testosterone; FAI, Free androgen index; SHBG, sex hormone binding globulin.

Multivariable logistic regression analyses (**Table 5**) showed that higher serum 25(OH)D levels were significantly correlated with lower risk of HA in women with PCOS [odds ratio (OR) = 0.985, 95% CI: 0.975 – 0.996, P = 0.007]. Although it was not a prominent association, this protective effect remained significant after adjusting age, BMI, WC, HOMA-IR and LDL in model 2 (OR = 0.986, 95% CI: 0.973 – 0.998, P = 0.026) and multiple confounders in model 3 (OR = 0.982, 95% CI: 0.969 – 0.995, P = 0.006). When exploring the risk for HA in 25(OH)D stratification, the OR value had a downward trend across 25(OH)D tertiles, but only in patients with sufficient 25(OH)D level (≥ 75 nmol/L), the risk of HA decreased with the increase of 25(OH)D level in the unadjusted model (OR = 0.224, 95% CI: 0.057 - 0.876, P = 0.032). However, this correlation became insignificant in model 2 (P = 0.123) and model 3 (P = 0.109).

**Table 5:** Association between 25(OH)D and HA stratified by 25(OH)D levels.

	Model 1	Model 2	Model 3
	OR (95% CI)	OR (95% CI)	OR (95% CI)
	P-value	P-value	P-value
**25(OH)D**	0.985 (0.975, 0.996) 0.007	0.986 (0.973, 0.998) 0.026	0.982 (0.969, 0.995) 0.006
**25(OH)D**			
** <50 nmol/L**	Reference	Reference	Reference
** ≥50, <75 nmol/L**	0.948 (0.574, 1.567) 0.836	0.878 (0.491, 1.572) 0.662	0.829 (0.450, 1.529) 0.548
** ≥75 nmol/L**	0.224 (0.057, 0.876) 0.032	0.299 (0.064, 1.389) 0.123	0.258 (0.049, 1.356) 0.109
**P for trend**	0.127	0.189	0.138

Model 1, no covariates were adjusted.

Model 2, Age, BMI, WC, HOMA-IR, LDL were adjusted.

Model 3, Age, BMI, WC, HOMA-IR, HbA1c, hs-CRP, ALT, AST, γ-GT, UA, TG, Tch, HDL, LDL, TSH, LH, FSH and E2 were adjusted.

### Subgroup Analyses

**Figure 1:**
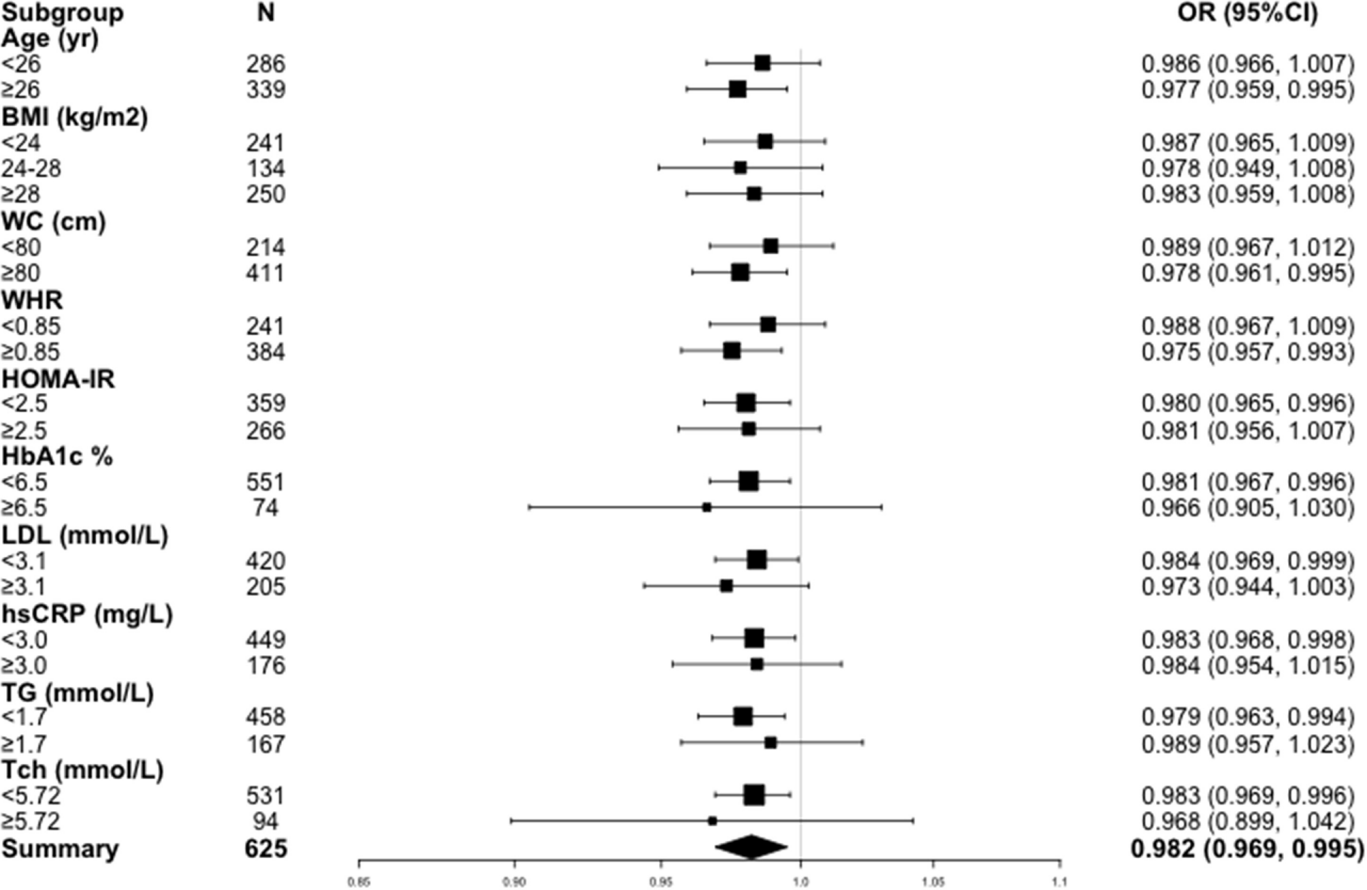
Odds ratios (ORs) for hyperandrogenemia in women with PCOS in subgroups. BMI, body mass index; WC, waist circumference, WHR, waist-hip circumference ratio; HOMA-IR, homeostasis model assessment of insulin resistance; HbA1c, hemoglobin A1c; LDL, low-density lipoprotein; hs-CRP, high-sensitivity C-reactive protein; TG, triglyceride; Tch, total cholesterol.

Sensitivity analysis was performed to confirm the robustness of the results by stratifying confounders (**Figure 1**). All analyses were adjusted for demographic factors (age, BMI and WC), metabolic biomarkers (HOMA-IR, HbA1c, hs-CRP, ALT, AST, γ-GT, UA, TG, Tch, HDL and LDL) and hormones (TSH, LH, FSH and E2). A consistent pattern was observed across subgroups. Notably, higher 25(OH)D levels were significantly associated with lower risks of HA among patients with age ≥ 26 years, WC ≥ 80 cm, WHR ≥ 0.85, HOMA-IR < 2.5, HbA1c < 6.5%, LDL < 3.1 mmol/L, TG < 1.7 mmol/L, Tch < 5.72 mmol/L and hs-CRP < 3.0 mg/L.

**Figure 2:**
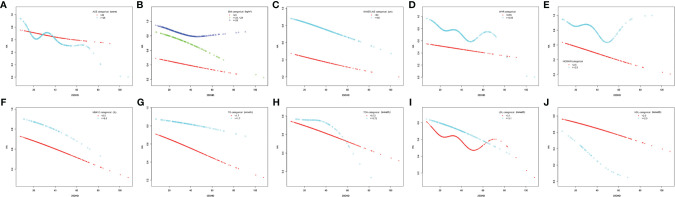
Association between 25(OH)D levels (nmol/L) and hyperandrogenemia in subgroups. **(A)** Age (years) (< 26; ≥ 26), **(B)** BMI (kg/m^2^) (< 24; 24 – 28; ≥ 28), **(C)** waistline (cm) (< 80; ≥ 80), **(D)** WHR (< 0.85; ≥0.85), **(E)** HOMA-IR (< 2.5; ≥ 2.5), **(F)** HbA1c (%) (< 6.5; ≥ 6.5), **(G)** TG (mmol/L) (< 1.7; ≥ 1.7), **(H)** Tch (mmol/L) (< 5.72; ≥ 5.72), **(I)** LDL (mmol/L) (< 3.1; ≥ 3.1) and **(J)** HDL (mmol/L) (< 2.0; ≥ 2.0).

Smooth curve fittings were then used to address the relationship between 25(OH)D and HA in the subgroup analyses stratified by age, BMI, WC, WHR, HOMA-IR, HbA1c, TG, Tch, LDL and HDL (**Figure 2**). As is shown, after adjusting multiple confounders (age, BMI, WC, HOMA-IR, HbA1c, hs-CRP, ALT, AST, γ-GT, UA, TG, Tch, HDL, LDL, TSH, LH, FSH and E2), a significant linear and negative correlation between 25(OH)D and HA was found in patients with BMI 24 ~ 28 kg/m^2^ (P = 0.038), WC ≥ 80 cm (P = 0.018), HOMA-IR < 2.5 (P = 0.018), HbA1c < 6.5% (P = 0.014), TG < 1.7 mmol/L (P = 0.007), Tch < 5.72 mmol/L (P = 0.011) and HDL < 2.0 mmol/L (P = 0.014), while a significant nonlinear correlation was observed in patients with WHR ≥ 0.85 (P = 0.037).

As vitamin D deficiency accounts for the majority of women with PCOS, we then evaluated the risk factors for HA in these patients. Data are shown in **Table 6**. Increased BMI, WC, WHR, HOMA-IR, HbA1c, LDL, hs-CRP and TG were regarded as risk factors of HA in patients with deficient vitamin D in unadjusted model (OR = 1.704 – 7.443, P < 0.01). After adjusting multiple confounders (age, BMI, WC, HOMA-IR, HbA1c, hs-CRP, ALT, AST, γ-GT, UA, TG, Tch, HDL, LDL, TSH, LH, FSH and E2), patients with age ≥ 26 years had lower risks of HA than those with age < 26 years (OR = 0.611, 95% CI = 0.389 - 0.958, P = 0.032), while patients with BMI 24 - 28 kg/m^2^ had higher risk of HA than those with BMI < 24 kg/m^2^ (OR = 2.202, 95% CI = 1.130 - 4.293, P = 0.020).

**Table 6:** Multinomial logistic regression model of risk factors for HA in PCOS women with vitamin D deficiency.

	Unadjusted			Adjusted*		
	OR	95% CI	P-value	OR	95% CI	P-value
**Age (years)**						
** <26**	1.000	Reference		1.000	Reference	
** ≥26**	0.943	0.660, 1.347	0.746	0.611	0.389, 0.958	0.032
**BMI (kg/m^2^)**						
** <24**	1.000	Reference		1.000	Reference	
** ≥24, <28**	4.443	2.678, 7.371	<0.001	2.202	1.130, 4.293	0.020
** ≥28**	7.443	4.708, 11.768	<0.001	1.137	0.467, 2.766	0.778
**WC (cm)**						
** <80**	1.000	Reference		1.000	Reference	
** ≥80**	5.550	3.759, 8.193	<0.001	1.553	0.843, 2.860	0.158
**WHR**						
** <0.85**	1.000	Reference		1.000	Reference	
** ≥0.85**	3.465	2.389, 5.027	<0.001	1.437	0.889, 2.323	0.139
**HOMA-IR**						
** <2.5**	1.000	Reference		1.000	Reference	
** ≥2.5**	4.644	3.080, 7.003	<0.001	1.147	0.658, 1.998	0.628
**HbA1c (%)**						
** <6.5**	1.000	Reference		1.000	Reference	
** ≥6.5**	3.081	1.529, 6.211	0.002	1.870	0.807, 4.334	0.144
**LDL (mmol/L)**						
** <3.1**	1.000	Reference		1.000	Reference	
** ≥3.1**	1.704	1.148, 2.530	0.008	0.796	0.407, 1.559	0.507
**hs-CRP (mg/L)**						
** <3.0**	1.000	Reference		1.000	Reference	
** ≥3.0**	3.338	2.090, 5.333	<0.001	0.842	0.463, 1.532	0.573
**TG (mmol/L)**						
** <1.7**	1.000	Reference		1.000	Reference	
** ≥1.7**	3.200	1.988, 5.150	<0.001	0.962	0.516, 1.792	0.902
**Tch (mmol/L)**						
** <5.72**	1.000	Reference		1.000	Reference	
** ≥5.72**	1.482	0.876, 2.507	0.143	0.619	0.264, 1.451	0.270

WC, waist circumference; WHR, waist-hip circumference ratio; LDL, low-density lipoprotein; hs-CRP, high-sensitivity C-reactive protein; TG, triglyceride; Tch, total cholesterol.

*Multiple confounders, including age, BMI, WC, HOMA-IR, HbA1c, hs-CRP, ALT, AST, γ-GT, UA, TG, Tch, HDL, LDL, TSH, LH, FSH and E2, were adjusted except the stratification variable itself.

## Discussion

This cross-sectional study of Chinese women with PCOS suggested that compared with controls, vitamin D levels were significantly lower in women with PCOS, especially in those with HA. An independent negative correlation between 25(OH)D levels and HA was noted in PCOS. Higher 25(OH)D levels were independently associated with lower risks of HA after adjusting demographic, metabolic and hormonal factors. Consistent results were observed in subgroup analyses stratified by age, BMI, WC, WHR, hs-CRP, HOMA-IR, HbA1c, LDL, TG and Tch. In addition, among PCOS women with vitamin D deficiency, age < 26 years and overweight were considered as independent risk factors of HA.

Vitamin D deficiency is regarded as the most common nutritional deficiency worldwide, with a prevalence between 58% and 91% of subfertile women reported by different studies (14). In line with this, our study reported a prevalence of vitamin D deficiency in 82.95% of controls and 86.24% of Chinese women with PCOS. The prevalence of vitamin D deficiency in women of childbearing age in China deserves more attention. Previous observational studies have been inconclusive in addressing vitamin D status in women with PCOS (14, 27, 28). A large study involving 639 women with PCOS and 449 controls reported that anovulatory women with PCOS had lower vitamin D levels than healthy controls (29). Consistent with these findings, our study reported lower serum 25(OH)D levels in Chinese women with PCOS compared with controls.

Accumulating evidence suggested an important role of vitamin D in regulating female fertility (17). Given that PCOS and vitamin D deficiency have overlapping metabolic features (15, 16), and based on the fact that vitamin D appears to influence aromatase activity in the ovaries which affects testosterone concentrations (30), the link between vitamin D and hyperandrogenism in PCOS was investigated in several studies. However, conflicting findings were reported with respect to this correlation at present. Meta-analysis conducted by Menichini et al. observed the beneficial effects of high-dose vitamin D supplementation (4000 IU) on total testosterone, SHBG and FAI (31). Nevertheless, the cross-sectional study by Mesinovic et al. found no associations between vitamin D metabolites and androgens in women with PCOS (30). Besides, Gallea et al. found that in subjects with PCOS, seasonal serum 25(OH)D levels seem to be related to insulin and body weight, but not hyperandrogenism (32). The differences in the places of residence, life styles, ethnic of included subjects, the sample size and the season of the studies conducted may contribute to this inconsistence. In our study, although we did not observe a prominent association between vitamin D and HA, an independent negative correlation between 25(OH)D levels and HA was noted in PCOS after adjusting demographic, metabolic and hormonal factors. We have also observed that as 25(OH)D levels increased, the risk of HA in PCOS had a downward trend, although it did not reach a statistical difference. It might attribute to the fact that as patients with insufficient and sufficient vitamin D accounted for only a small part of women with PCOS in this study, the protective effect of 25(OH)D on HA may not be fully demonstrated. Further in-depth research is needed to clarify the undeniably complex relationship between vitamin D status and HA in women with PCOS.

PCOS is closely related to characteristics of metabolic syndrome, including abnormal glucose metabolism, IR, and obesity (33). A meta-analysis demonstrated that 30 - 40% of women with PCOS were accompanied by IR and compensatory hyperinsulinaemia, and that 10% of them will develop type 2 diabetes mellitus (34). In addition, compared with healthy women, PCOS women were more prone to dyslipidaemia, such as higher TG and LDL, and lower HDL (35). In line with these findings, our study also reported higher WC, HOMA-IR, TG, Tch, LDL levels and lower HDL levels in women with PCOS, especially in those with HA. Mounting evidence have demonstrated the link between vitamin D status and metabolic health (28). According to our subgroup analyses stratified by multiple metabolic factors, the significant negative correlation between 25(OH)D and HA was observed in PCOS women with abdominal obesity but normal glucose and lipid status, suggesting the importance of sufficient vitamin D status in the early stage of PCOS before the disturbance of glucose and lipid metabolism.

When further analyzing the features of PCOS women with deficient vitamin D levels, we observed an increased risk of HA in overweight patients with age < 26 years. Previous studies have demonstrated the changes of phenotype of PCOS during the aging process (36) and reported a negative correlation between androgen markers with age (37), which may partly explain our finding regarding to the higher risks of HA in younger patients with PCOS. Besides, as a major determinant of metabolic health and long-term complications in PCOS, weight-gain appears to worsen biochemical HA (38, 39). However, compared with those with BMI < 24 kg/m^2^, higher risk of HA was only found in overweight patients, not obese patients with PCOS in our study. Possible reasons may as follow: First, when comparing androgens in BMI stratifications, similar testosterone levels but significantly different DHEAS levels were found, and DHEAS levels were especially higher in overweight patients, indicating the possibly different pathogenesis of HA in overweight PCOS women. Second, the directionality of the relationship between PCOS and obesity remains unclear (27). On one hand, excess body weight and abdominal obesity are very common in PCOS (40); on the other hand, obesity, particularly developing during the adolescence, may be responsible for the development of PCOS. Obese women with PCOS from different origins may have different androgen levels, which may affect our final conclusion about the relationship between BMI and HA. Third, as BMI includes not only adipose tissues but also muscle mass and body water that can vary in different populations (41), our future studies that investigate specific aspects of body composition may offer more detailed insights into the relationship between BMI and HA in PCOS. Therefore, if there is vitamin D deficiency, women with PCOS that have higher BMI levels with age < 26 years may be prioritized for HA assessment.

Oral contraceptive, androgen receptor blocker, 5α-reductase inhibitor and glucocorticoid are the most common anti-androgen pharmacologic therapies in PCOS at present (42). However, there are often cases in clinical practice where there are drug contraindications or the therapeutic effects of these drugs are not very satisfied. There may be a place for vitamin D supplementation in the management of HA in PCOS due to its potential therapeutic efficacy, safety and acceptability, but current evidence is limited. Based on our results, evaluation of serum 25(OH)D levels and sufficient vitamin D supplementation is especially recommended for younger overweight women with HA. Further investigations *via* long-term randomized clinical trials are warranted to confirm our findings.

There are several limitations in our study to be illustrated. First, since our study was a cross-sectional study, the causality of the association between hypovitaminosis D and hyperandrogenism cannot be established. Meanwhile, the generalizability may be geographically restricted as our study was a single-center study and the research population was limited in Eastern China. Further long-term and multi-center studies with large sample size are needed to explore the causal relationship between vitamin D and hyperandrogenism. Second, variables including sunlight exposure status and season for detection of serum 25(OH)D levels were lacking in our study. However, considering the large sample size of our current study, it is very regrettable that we really can’t retain all samples in the same season. Moreover, other study found serum vitamin D deficiency was common in both winter and summer among child-bearing period healthy women in Beijing (43). In our future research design, the potential impact of seasonal factors on the concentration and effect of vitamin D will be more comprehensively considered. Third, although ECLIA is an efficient and accurate method for measuring serum 25(OH)D, the liquid chromatography-mass spectrometry (LC-MS) assay is needed in the future to verify the associations between serum 25(OH)D levels and hyperandrogenism in PCOS. However, a series of studies have demonstrated that ECLIA was comparable to LC-MS/MS in detection of serum 25(OH)D levels and very suitable for routine clinical use (44–46).

## Conclusions

In conclusion, our study reported lower vitamin D levels in Chinese women with PCOS, especially in those with HA. An independent negative correlation between 25(OH)D and HA was noted in PCOS. For PCOS women with vitamin D deficiency, females that have higher BMI with age < 26 years may be prioritized for HA assessment. Our study provides more evidence for linking vitamin D with HA in PCOS.

## Data Availability Statement

The original contributions presented in the study are included in the article/supplementary material. Further inquiries can be directed to the corresponding author.

## Ethics Statement

The studies involving human participants were reviewed and approved by the Ethical Committees of Renji Hospital, School of Medicine, Shanghai JiaoTong University. The patients/participants provided their written informed consent to participate in this study.

## Author Contributions

CS, Y-cZ, JY, YZ, Y-yW, NL, and JC collected the data. WL and TT designed the study. CS and Y-cZ analyzed the data. CS and TT wrote the draft of this article. All authors made critical revisions of the manuscript. All authors contributed to the article and approved the submitted version.

## Funding

This study was supported by the National Natural Science Foundation of China (82170807), Shanghai Sailing Program (21YF1425300), National Natural Science Foundation of China (81800779), the Medical Guidance Science and Technology Support Projects of Shanghai Municipal Science and Technology Commission (18411968700), and the Natural Science Foundation of Shanghai (12ZR1417800).

## Conflict of Interest

The authors declare that the research was conducted in the absence of any commercial or financial relationships that could be construed as a potential conflict of interest.

## Publisher’s Note

All claims expressed in this article are solely those of the authors and do not necessarily represent those of their affiliated organizations, or those of the publisher, the editors and the reviewers. Any product that may be evaluated in this article, or claim that may be made by its manufacturer, is not guaranteed or endorsed by the publisher.
